# Less than full circumferential fusion of a tibial nonunion is sufficient to achieve mechanically valid fusion - Proof of concept using a finite element modeling approach

**DOI:** 10.1186/1471-2474-15-434

**Published:** 2014-12-15

**Authors:** Thorsten Tjardes, Michael Roland, Robin Otchwemah, Tim Dahmen, Stefan Diebels, Bertil Bouillon

**Affiliations:** Department of Trauma and Orthopedic Surgery, University of Witten/Herdecke, Faculty of Health - School of Medicine, Cologne Merheim Medical Center, Ostmerheimerstr. 200, 51109 Cologne, Germany; Saarland University, Chair of Applied Mechanics, 66123 Saarbruecken, Germany; German Research Center for Artificial Intelligenz, Campus D3 2, Stuhlsatzenhausweg 3, 66123 Saarbruecken, Germany

## Abstract

**Background:**

Although minimally invasive approaches are widely used in many areas of orthopedic surgery nonunion therapy remains a domain of open surgery. Some attempts have been made to introduce minimally invasive procedures into nonunion therapy. However, these proof of concept studies showed fusion rates comparable to open approaches never gaining wider acceptance in the clinical community. We hypothesize that knowledge of mechanically relevant regions of a nonunion might reduce the complexity of percutaneous procedures, especially in complex fracture patterns, and further reduce the amount of cancellous bone that needs to be transplanted. The aim of this investigation is to provide a proof of concept concerning the hypothesis that mechanically stable fusion of a nonunion can be achieved with less than full circumferential fusion.

**Methods:**

CT data of an artificial tibia with a complex fracture pattern and anatomical LCP are converted into a finite element mesh. The nonunion area is segmented. The finite element mesh is assigned mechanical properties according to data from the literature. An optimization algorithm is developed that reduces the number of voxels in the non union area until the scaled von Mises stress in the implant reaches 20% of the maximum stress in the implant/bone system that occurs with no fusion in the nonunion area at all.

**Results:**

After six iterations of the optimization algorithm the number of voxels in the nonunion area is reduced by 96.4%, i.e. only 3.6% of voxels in the non union area are relevant for load transfer such that the von Mises stress in the implant/bone system does not exceed 20% of the maximal scaled von Mises stress occurring in the system with no fusion in the non union area at all.

**Conclusions:**

The hypothesis that less than full circumferential fusion is necessary for mechanical stability of a nonunion is confirmed. As the model provides only qualitative information the observed reduction of fusion area may not be taken literally but needs to be calibrated in future experiments. However this proof of concept provides the mechanical foundation for further development of minimally invasive approaches to delayed union and nonunion therapy.

**Electronic supplementary material:**

The online version of this article (doi:10.1186/1471-2474-15-434) contains supplementary material, which is available to authorized users.

## Background

The principles of minimally invasive surgery have changed the face of many fields of surgery. In orthopedic trauma surgery it has decreased the damage to soft tissues, minimized scaring, reduced postoperative pain and improved recovery time. These aims have basically been reached by reducing the invasiveness of the instruments and surgical techniques, e.g. minimally invasive plate osteosynthesis (MIPO) or percutaneous pedicle screw instrumentation in spine surgery. The complementary strategy, i.e. reducing the extent of the procedure, has so far not been pursued systematically, as the extent of an instrumentation is usually defined by the biomechanical demands of the injury itself.

In nonunion therapy open resection and autologous cancellous bone transplantation, which is a highly invasive in terms of disturbance of local vascularity has become the gold standard approach. However, in this field too several attempts to reduce the invasiveness of the procedure have been made. Kettunen et al. [1] replaced a cylindrical cut at the site of the nonunion by a similar size bone cylinder from the iliac crest and reported a union rate of 90% in 41 patients after 13 weeks (range 10–48 weeks). Bhan et al. [2] percutaneously treated tibial delayed union and nonunion with autologous bone grafting using a specially devised milling cutter and achieved union in 18/21 patients. Similarly Maghsudi et al. [3] performed percutaneous bone grafting in 11 tibia nonunions after local resection of the nonunion using a 5.5 mm acromionizer. They achieved a healing rate of 88% and consider their technique as suitable for limited size bone defects. Johnson et al. [4] applied arthroscopic techniques to nonunion resection and bone grafting in a proof of concept study with nine patients achieving union in 8/9 patients. The highest degree of evidence was reported by Maneerit et al. [5] in a randomized trial comparing open to percutaneous bone grafting for tibial nonunion. Regarding the primary endpoint, i.e. union, non differences were found, while the percutaneous group was significantly better in terms of operation time and blood loss.

Given this evidence on minimally nonunion therapy and taking into account the load adaptive behavior described by Wolffs law as well as everyday experience from clinical medicine that full load bearing is possible in the absence of full circumferential fusion of a fracture it becomes evident that there is a gap of knowledge concerning the biomechanics of nonunions. Recently Lack et al. [6] reported that any bone bridging by four month in tibia OTA 42-A,B,C fractures separates fractures that heal without further intervention from those that progress to non union with an accuracy of 99% in fractures treated with intramedullary nailing. In the clinical context this evidence is very helpful in identifying those patients with a sufficient biological potency to complete fracture healing.

More important, the study presented by Lack et al. is the first clinical study to support the hypothesis that full circumferential fusion of a fracture is not a prerequisite for mechanical and thus functional stability in tibia fractures.

Taken together these observations support the hypothesis that the identification and targeted approach of mechanically relevant regions of a nonunion might reduce the complexity of percutaneous procedures, especially in complex fracture patterns,9 and further reduce the amount of cancellous bone that needs to be transplanted.

To approach this hypothesis the present work provides the general proof of concept that an individualized finite element based simulation of a tibia nonunion which is subjected to physiologic loading, requires less than full circumferential fusion to achieve physiologic load bearing capacity.

## Methods

### Preparation of the fracture model

An AO Typ 42–B1.1 [7] was implemented in a tibia model (Sawbones Europe, Malmø, Sweden). The surface of the sawbone was treated with zinc spray to increase radiographic visibility. The fracture was fixed with a 14 hole titanium distal tibia locking compression plate (anatomical LCP, Synthes, Oberdorf, Switzerland). A computed tomography scan of the fracture model was acquired (Somatom Definition Flash, Siemens, Germany).

### Mesh generation and image processing

The CT raw data are directly converted into computational meshes using standardized image processing algorithms. 726 single CT images in the unit12 data format, these twelve–digit binary numbers can be mapped linearly to the Hounsfield scale. The slice thickness of the CT data set is constant with a distance of 0.6 mm between two pictures. The images are square–cutted with 512 pixels as height and width. The pixel spacing is also equal inside all images with rounded off 0.318 mm.

Image segmentation is based on anisotropic diffusion algorithms for image processing [8] in combination with edge–preserving regularization and edge detection techniques [9, 10]. Then the image data was merged to a finite element mesh which contains nearly the possible maximum of the information from the computed tomography, i.e. all segmented pixels are transformed to voxels according to the slice thickness of the CT. The grayscale value of each pixel will be mapped on the barycenter of the corresponding voxel. Finally the voxels of the nonunion area are marked in a semi–automatic segmentation process. For numerical simulation the material properties of the nonunion area are set to the material properties of soft tissue [11].

### Coarsening algorithm

**Figure 1:**
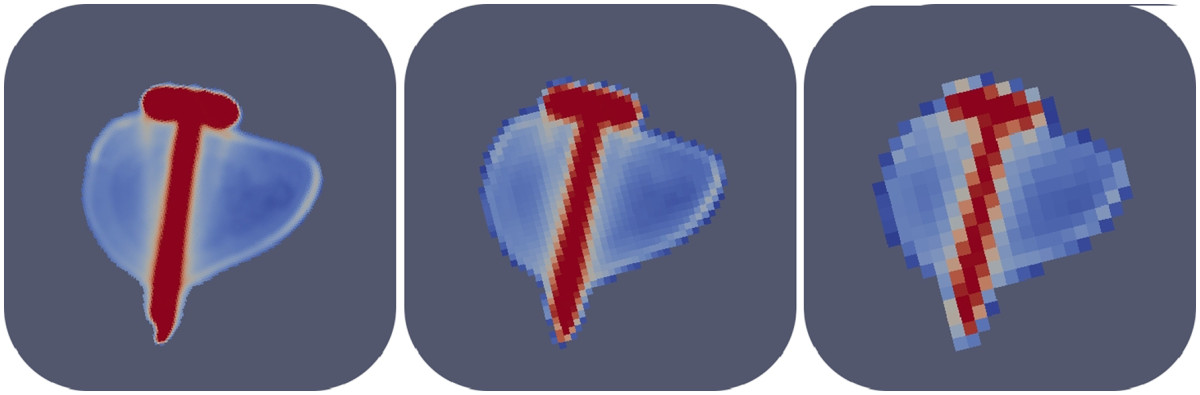
**Screw bone interface after repeated runs of the coarsening algorithm.** Left panel corresponding to the level 1 finite element mesh, middle panel corresponding to the level 4 finite element mesh, and right panel corresponding to the level 7 finite element mesh.

To accelerate the computation and to require less memory, the mesh was coarsened with respect to the volume fractions of the particular materials and their properties. The coarsening algorithm operates on the 2D image plane. For each level of coarsening, the algorithm moves a level × level–pixel window through the segmented images and combines the pixels inside the window to a new larger pixel (Table 1). To determine the material properties of the new pixel, the material properties inside the window were averaged by an arithmetic mean and thereafter cutted with an appropriate threshold value.To further reduce the expense of computer memory, the epiphysis of the mesh was cut proximally and distally resulting in a further reduction of the number of mesh cells of approximately 30 percent (Figure 1).

**Table 1:** **Optimisation algorithm in pseudo code**

Optimisation algorithm	
1:	**initialize** the algorithm:
2:	compute the worst case scenario
3:	set the material parameters of each nonunion area mesh cell to soft tissue
4:	compute the von Mises stress for the tibia
5:	**search** for the maximum stress value
6:	**define** max:=maximum stress value
7:	compute the initial scenario
8:	set the material parameters of each nonunion area mesh cell to cortical bone
9:	compute the von Mises stress for the tibia
10:	**define** the stress threshold
11:	**define** the stop_criterion
12:	**repeat**
13:	**if** von Mises stress of nonunion area mesh cell < threshold x max
14:	set material parameter of each nonunion area mesh cell from cortical bone to soft tissue

Beginning in a state of complete union fracture area mesh cells with von Mises stress < threshold x max are identified. The material parameters for these cells are set from cortical bone to soft tissue. After one cycle over all fracture area mesh cells, the new material parameters are used as starting point for the next optimization run.

### Optimization algorithm

The optimization algorithm iteratively simulates cyclic loading of the bone/implant system and calculates the von Mises stress in the bone/implant system. After each simulation the fusion area in the fracture is reduced incrementally until a predefined stop criterion (Table 1), i.e. 20% of the maximum von Mises stress arising in the current step of the algorithm is reached. With the number of unfilled area increasing with every run of the optimization algorithm, the maximum of the von Mises stress in the remaining area and the implant constantly increases.

The fusion area that remained when the stop criterion was reached defined the minimum necessary fusion area that needs to result from cancellous bone transplantation to achieve mechanical stability.

The boundary conditions for the algorithm were given by either complete consolidation or by complete nonunion in the fracture area. Initially a ‚worst case’ scenario is calculated, i.e. the material parameters of the region marked as nonunion area during the segmentation process are set to the parameters of soft tissue [11]. The optimization process was started from a state of complete union, i.e. the material parameters of the nonunion area were set to cortical bone.

### Numerical simulation

To derive an empirical elasticity–bone density relationship for the artificial bone, the grayscale values of the CT data were mapped to the Hounsfield scale and thereafter to the local bone properties [12–14]. This was done in analogy to the approach for real bones by means of a mapping based on a power law relationship and a calibration phantom [15–17]. The apparent density as densitometric measure was used to compute the real density, the Young’s modulus and the Poisson’s ratio.

Numerical simulation runs are performed iteratively at step 15 (Table 2) of the optimization algorithm. During the simulation a cyclic load equivalent to an 80 kg person was applied in a cranio-caudal direction.

**Table 2:** **List of all coarsening levels, height and width of one image, and the total number of voxels in the corresponding finite element mesh**

Coarsening process		
Level	[Height x level]	Number of voxels
**1**	[512 × 512]	7.545.550
**2**	[256 × 256]	1.930.582
**3**	[170 × 170]	878.539
**4**	[128 × 128]	505.844
**5**	[102 × 102]	331.259
**6**	[85 × 85]	235.174
**7**	[73 × 73]	176.488
**8**	[64 × 64]	138.033

## Results

**Figure 2:**
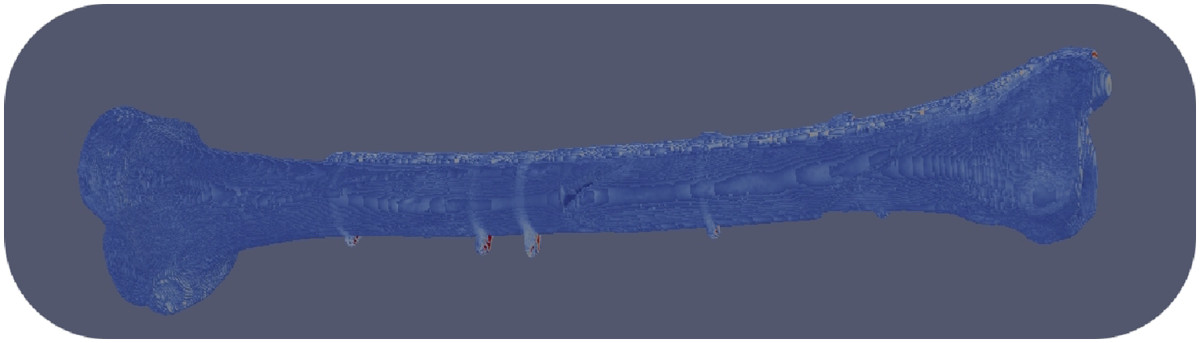
**Finite element mesh generated from the computed tomography data with approximately 7.5 million mesh cells ( = voxels).**

Conversion of the CT raw data into a finite element model yielded an finite element mesh with approximately 7.5 million mesh cells, i.e. voxels (Figure 2).

### Image segmentation

**Figure 3:**
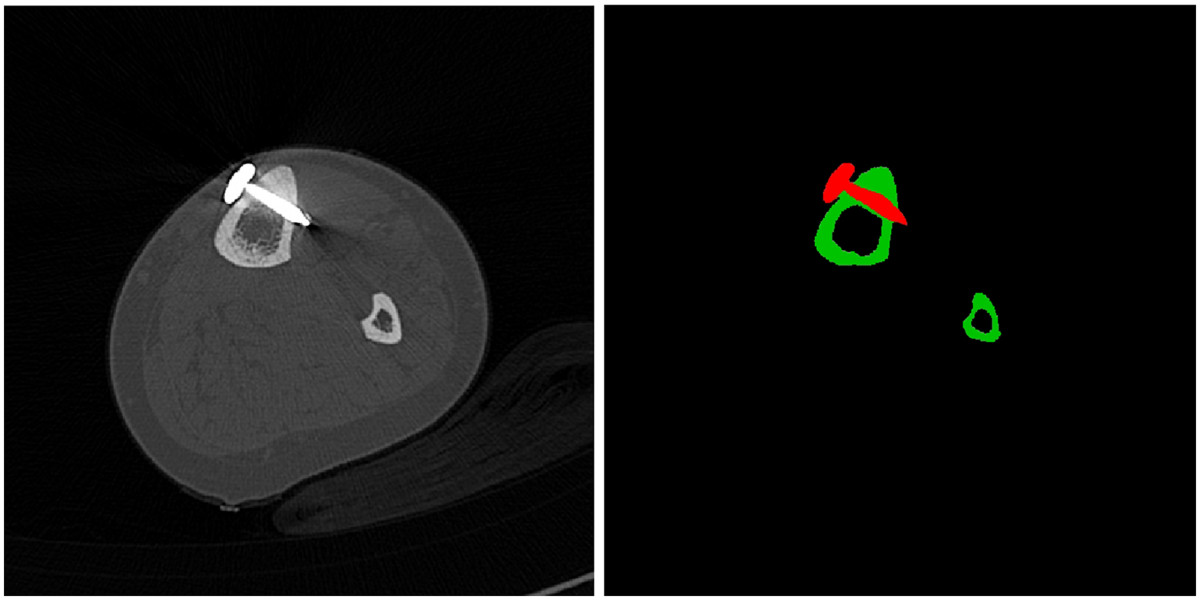
**Original computed tomography image (left); results of the segmentation process (right).**

**Figure 4:**
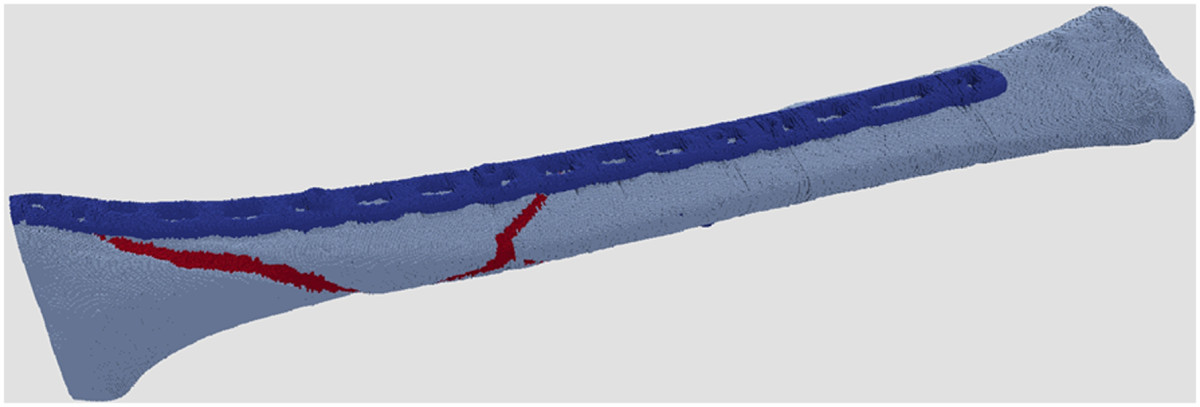
**Finite element mesh of the tibia with titanium implant (blue) and nonunion area (red) after completion of the segmentation process.**

Image segmentation separated the soft tissue signals from the bone and the implant part of the image data (Figure 3). The area of the pseudarthrosis were identified and marked in a semi automated segmentation process (Figure 4).

### Coarsening

The coarsening algorithm reduced the number of voxels from 7.545.550 voxels to 92.418 voxels after ten iterations of the coarsening algorithm (Table 2), i.e. the number of voxels was reduced by 98%.

The result of the coarsening process in the vicinity of the bone screw interface is shown in Figure 1. The panels correspond to the levels 1, 4 and 7 of the coarsening algorithm (Table 2). The structures at the bone screw interface became increasingly blurred at their edges. The profile of the screw was getting slightly thinner due to averaging and thresholding of the material properties with every iteration of the coarsening algorithm. However, comparing the image of the level 4 mesh with the level 1 mesh image demonstrates that a 93% reduction of pixels still gives a good approximation of the level 1 image. The following numerical simulations are thus performed on level 4 meshes corresponding to 505.844 voxels.To reduce the computational cost, the epiphysis of the mesh was cut proximally and distally resulting in a 30% reduction of the mesh cells (Figure 4).

After coarsening the mesh contains 381.343 nodes and 342.648 mesh cells including 76.122 boundary elements with 1.144.029 degrees of freedom for linear finite elements and to 8.684.082 degrees of freedom for quadratic finite elements.

### Numerical simulation and optimization algorithm

**Figure 5:**
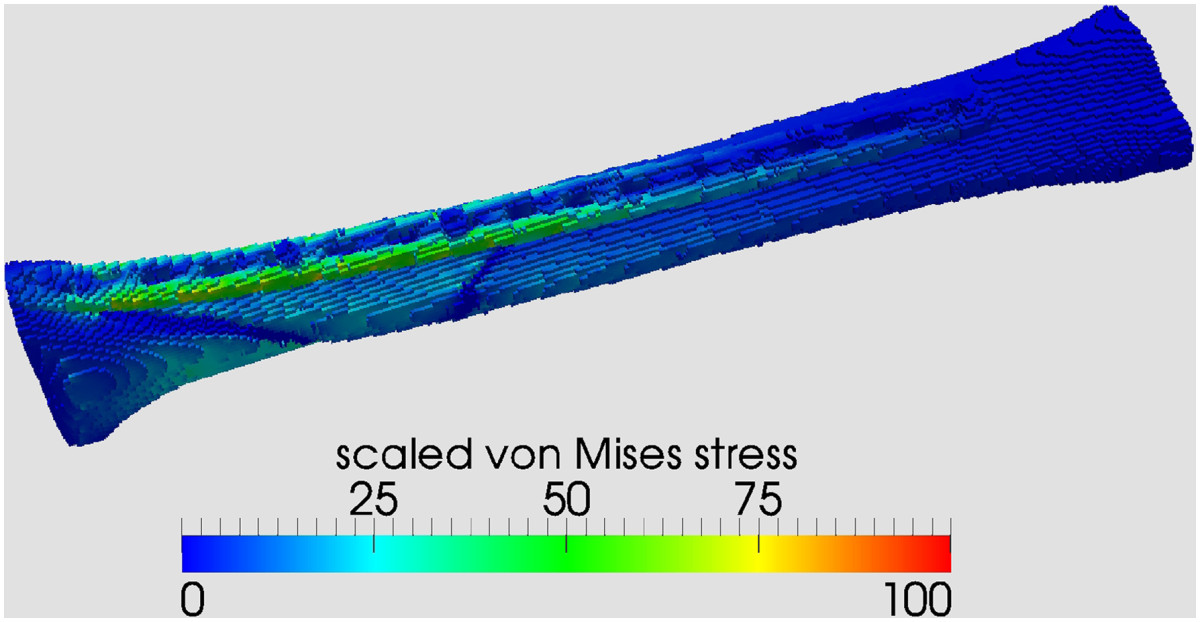
**Numerical result for axial weight bearing of an 80 kg person with no osseous consolidation in the nonunion area (‘worst case scenario’).**

The first simulation was run with the material properties in the nonunion area set to those of soft tissue (‚worst case scenario’) and an axial loading corresponding to a 80 kg person. Consequently the implant completely absorbs the von Mises stress while there is zero stress in the nonunion area (Figure 5). For ease of further calculations the total maximum von Mises stress of the worst case scenario of 2.25e7 Pa^-1^ was scaled to 100.

### Optimisation algorithm

The iterative application of the optimization algorithm, starting with material properties in the nonunion area similar to cortical bone, showed maximum scaled von Mises stress in step 1 as 79.64 (Table 3). Thus at least 50% of the nonunion area did not contribute to the stress distribution given the stop criterion of 80% of the maximal von Mises stress occurring with all nonunion area mesh cells set to the material properties of soft tissue.

**Table 3:** **Reduction of the nonunion area mesh cells in every step of the algorithm (row 1); maximum von Mises stress for every step of the algorithm (row 2)**

Detection of minimal fusion area							
**Step**	**0**	**1**	**2**	**3**	**4**	**5**	**6**
**Reduction [%]**	-	50.7	3.4	21.9	12.2	5.5	2.7

**Figure 6:**
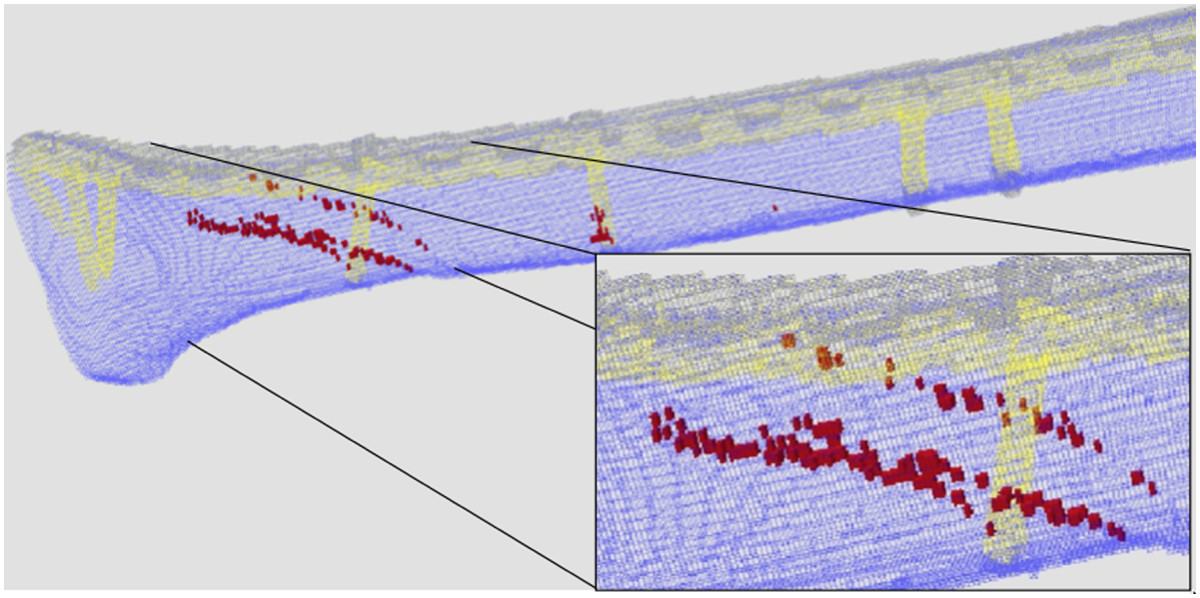
**Remaining parts of the nonunion area (red) necessary for load transfer within the boundary criterion after iterative runs of the optimisation algorithm.**

After six iterations of the algorithm and accepting slightly extended stop criteria in iteration steps 4–6 (Table 3), the number of nonunion area mesh cells necessary to keep the stress distribution within the boundary condition dropped to 3.7% of the total nonunion area (Figure 6).

## Discussion

Minimally invasive procedures have changed the face of many surgical procedures in the last decades. Although some attempts had been made to introduce minimally invasive techniques of treatment of nonunion [1–5] the gold standard remains open resection and autologous bone grafting. Based on the load adaptive properties of bone described by Wolffs law we hypothesized that each fracture has a distinct pattern of fusion across the nonunion that is sufficient to reach the endpoint of mechanically stable fracture union once bone remodeling has taken place and that this pattern is less than the full circumferential fusion of a nonunion usually attempted with autologous cancellous bone grafting.

Using a finite element model of a complex tibia fracture that was fixed with an anatomical locking plate an optimization algorithm was applied that, starting from a completely consolidated nonunion, reduced the number of finit element voxels in the nonunion area until the von Mises stress in the implant reached the stop criterion of 20% of the maximum von Mises stress arising in the current step of the algorithm. This procedure asymptotically reduced the number of voxels in the nonunion area participating in the load transfer such that the implant did not reach a critical loading condition by 96.4%. Thus the hypothesis that less fusion area than full circumferential fusion is necessary to achieve mechanical stability has been confirmed on a qualitative level.

The results are limited to a qualitative statement because a hybrid model was developed, i.e. a combination of an artificial bone that was matched with biomechanical parameters based on the literature data. This approach was considered appropriate in the context of a proof of concept study as the artificial bone does have cortical and cancellous bone components that can be differentiated on CT images with sufficient accuracy, such that the screw purchase in either cancellous bone or cortical bone is adequately mapped to the model.

The second limitation of the model is that the loading parameters are limited to axial loading of the model. This means that the kinematics of gait are not reproduced physiologically. Thus it can be expected that the fusion area to compensate for torsional forces that occur during the gait cycle is larger compared to the extrem reduction of mechanically necessary voxels in the nonunion area seen in the current experiment. Alternatively the distribution of the fusion area identified by the optimization algorithm might differ if the complete gait cycle is used during simulation, as the intensity of shear stress on the implant and the nonunion can be expected to increase.

The results obtained during the simulation process explicitly do not take into account dynamics of callus formation and modulation during weight bearing, i.e. Wolff’s law of load adaptive maturation of the bone is not part of the model at this stage. However, in the context of the present work an implementation of the dynamics of fracture healing was considered to increase the complexity of the model and the cost of computer calculation without providing validated information beyond a proof of concept.

Due to the limitations mentioned above a more sophisticated analysis of the mechanical behavior of the model system in terms a an in depth analysis of the stresses and strain occurring in the the implant and the bone will not add valid information at this point of model development. For this reason reason scaled von Mises stress adequately reflects the behavior of the system and serves as single control parameter for the optimization algorithm.

The stop criterion was set to the scaled value of 84%. This is a moderate and potentially reasonable increase of the stress peak. The optimization algorithm quickly converges to scaled von Mises stress of approximately 80% corresponding to a mechanically relevant pseudarthrosis area of 50% in the first step. In future models which more precisely address the issues described above a less dramatic but still relevant reduction of voxels in the nonunion area can be expected with 50-75% being a realistic target.

## Conclusions

The observations of the present study support a general reconsideration of the concept of delayed union and non union of fractures. The results of the present biomechanical analysis demonstrate that there might be an individual biomechanical solution for each non union such that, given local biological potency is intact, the endpoint of full circumferential fusion is not mandatory.

Complementary to these experimental findings is the work of Lack et al. [6] who, for the first time, provided clinical evidence that carries the aspects of the diamond concept [18] to the clinical arena.

There are several implications of these observations for future directions of research on minimally invasive treatment of nonunion. As the pattern of mechanically relevant fusion points in a nonunion is defined by the individual fracture a quantitative information can only be generated with models of individual fractures. To achieve this functioning work flows with low computational cost allowing simulation in real time need to be developed. Also simulation protocols closer to clinical reality, i.e. mimicking different rehabilitation schedules and more closely mimicking the kinematics of gait need to be implemented into the simulation algorithm. Last not least surgical instruments have to be developed that allow a safe and tissue sparing approach to the regions of the nonunion that need to be supported with cancellous bone transplantation.

It can be expected that the combination of quantitative individualized modeling and minimally invasive surgery will improve healing rates, reduce the perioperative morbidity, decrease secondary endpoints like blood loss, hospital stay postoperative pain.

## Authors’ original submitted files for images

Below are the links to the authors’ original submitted files for images. Authors’ original file for figure 1 Authors’ original file for figure 2 Authors’ original file for figure 3 Authors’ original file for figure 4 Authors’ original file for figure 5 Authors’ original file for figure 6
