# Supplementary figures and images for: Less than full circumferential fusion of a tibial nonunion is sufficient to achieve mechanically valid fusion - Proof of concept using a finite element modeling approach
Source: BMC Musculoskelet Disord. 2014 Dec 15;15:434. doi: 10.1186/1471-2474-15-434 (PMC4301034; doi:10.1186/1471-2474-15-434)

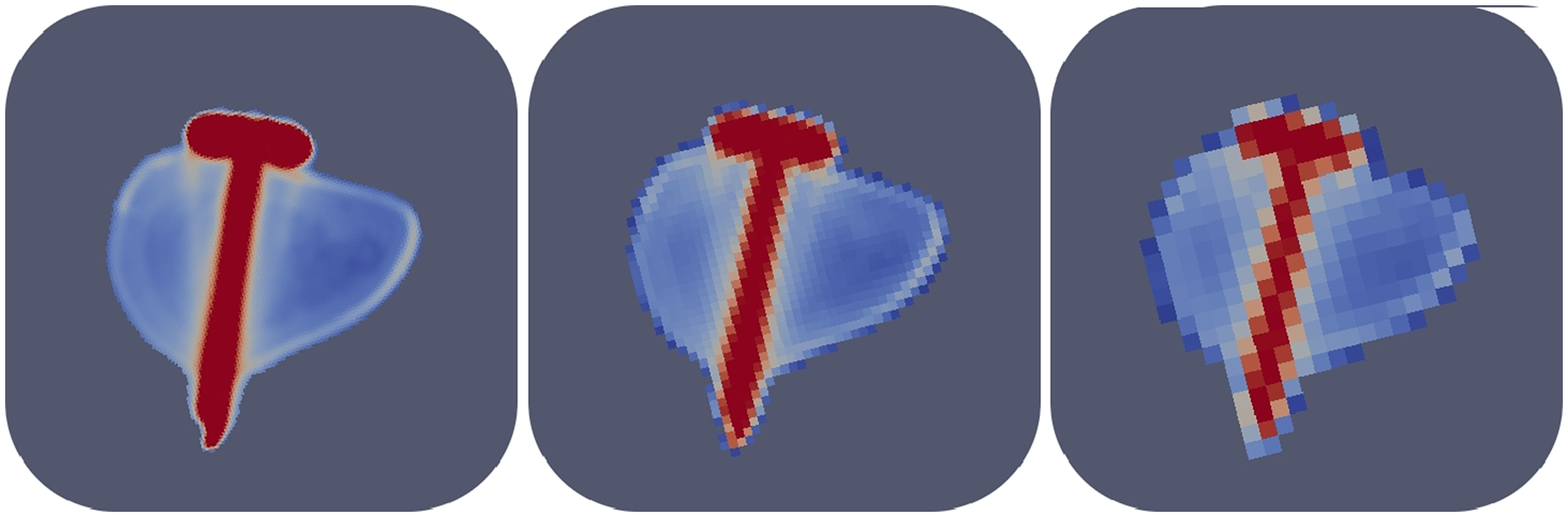

Supplement: Supplementary file 1 — Authors’ original file for figure 1 [file 12891_2014_2362_MOESM1_ESM.tiff]

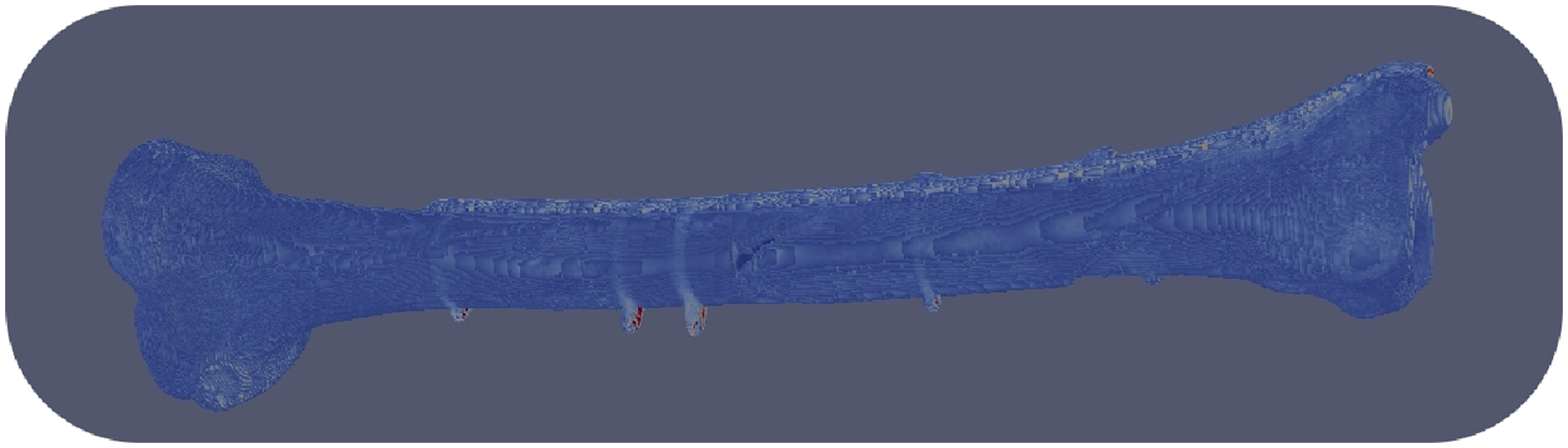

Supplement: Supplementary file 2 — Authors’ original file for figure 2 [file 12891_2014_2362_MOESM2_ESM.tiff]

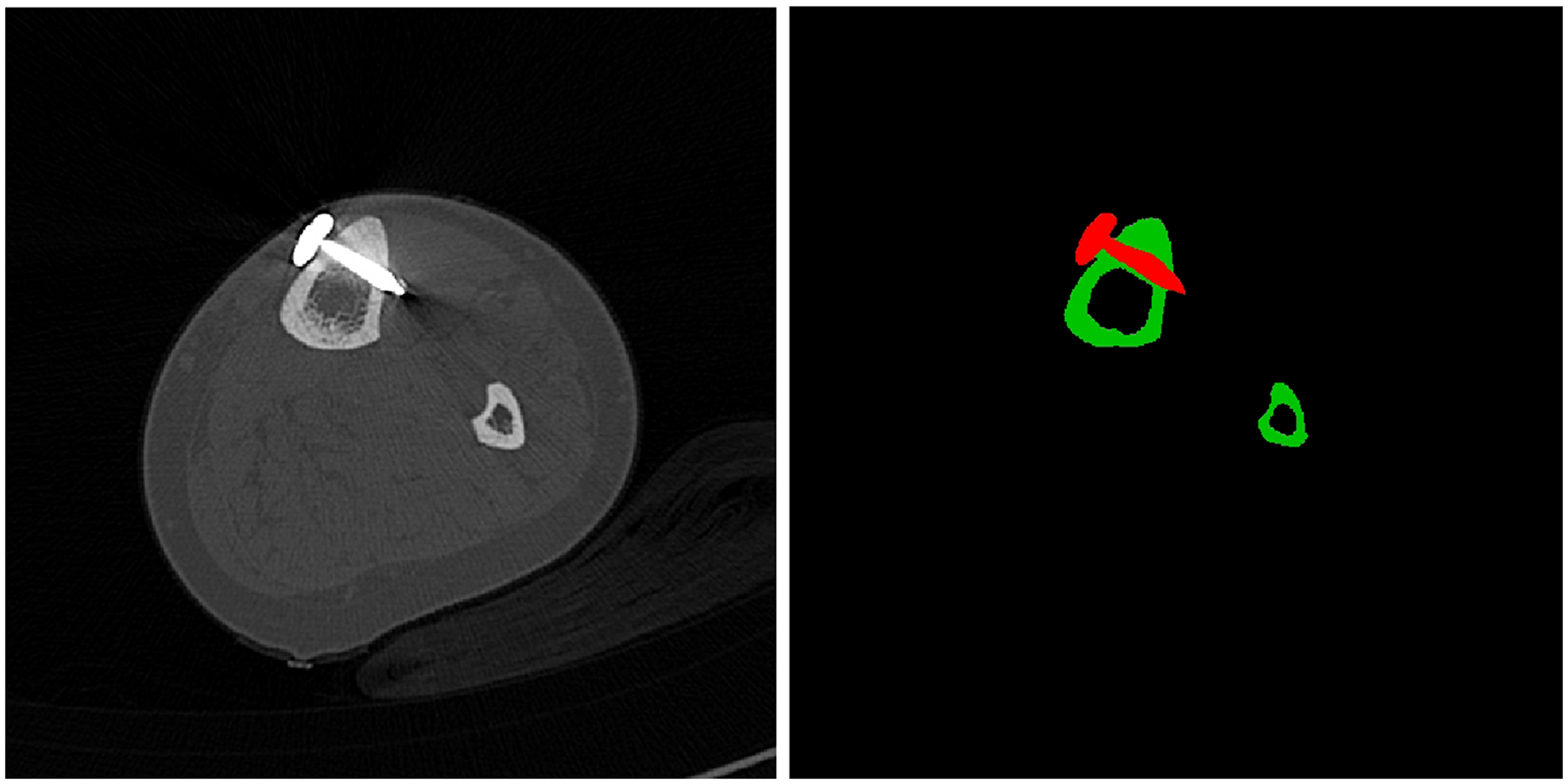

Supplement: Supplementary file 3 — Authors’ original file for figure 3 [file 12891_2014_2362_MOESM3_ESM.tiff]

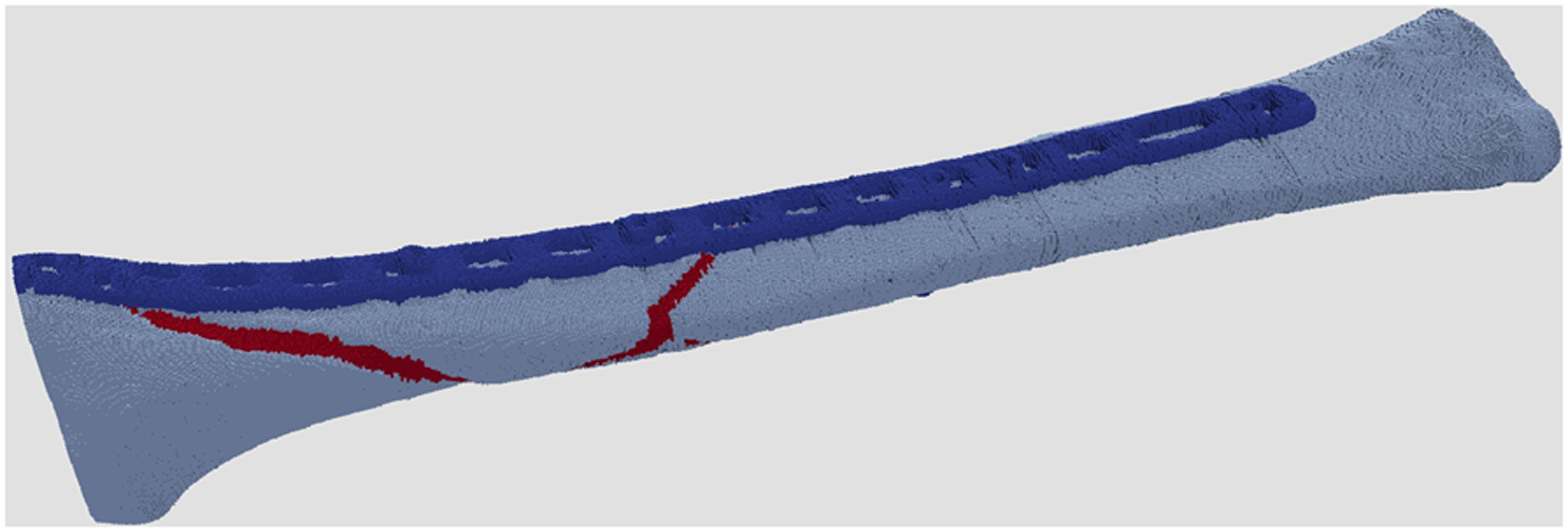

Supplement: Supplementary file 4 — Authors’ original file for figure 4 [file 12891_2014_2362_MOESM4_ESM.tiff]

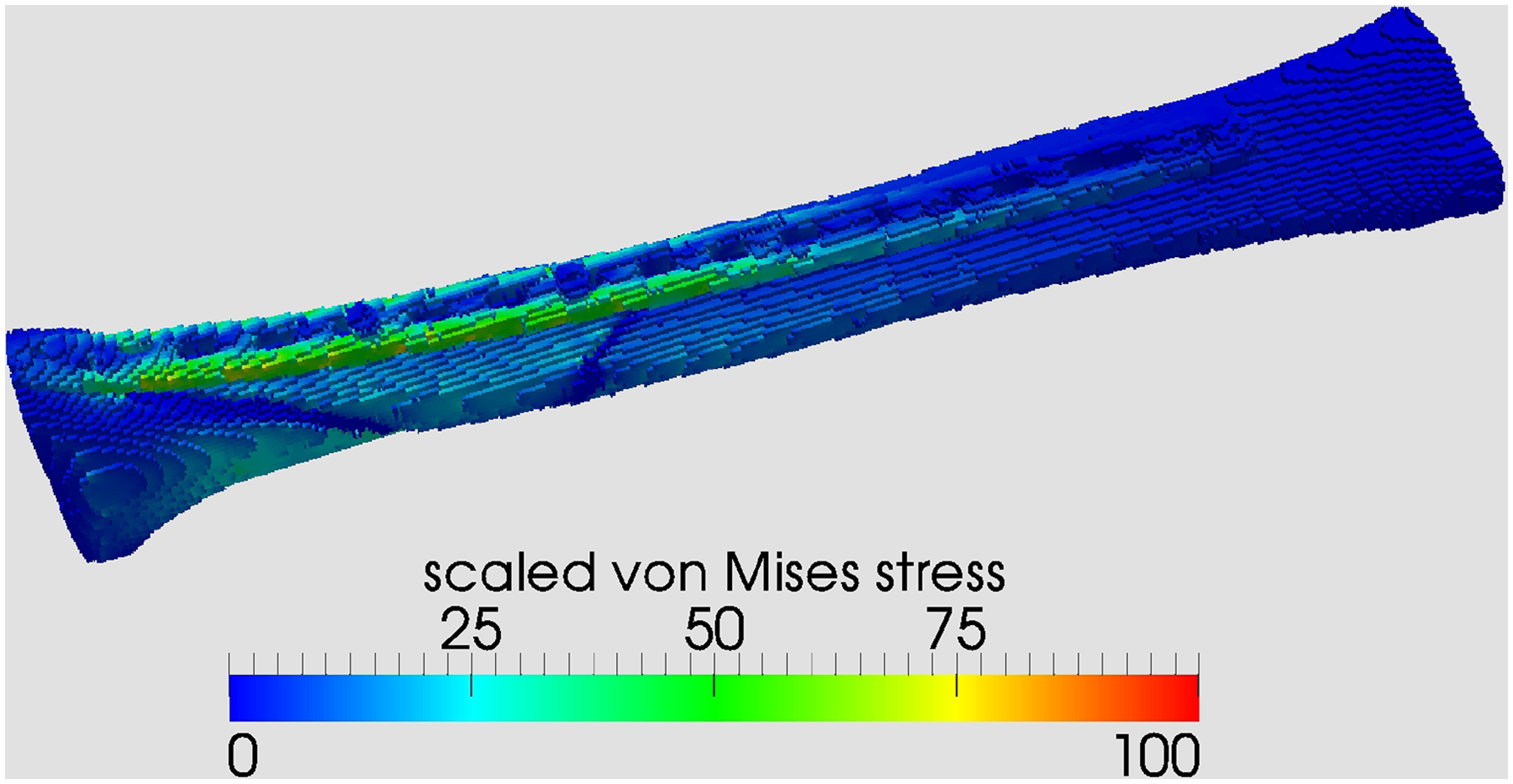

Supplement: Supplementary file 5 — Authors’ original file for figure 5 [file 12891_2014_2362_MOESM5_ESM.tif]

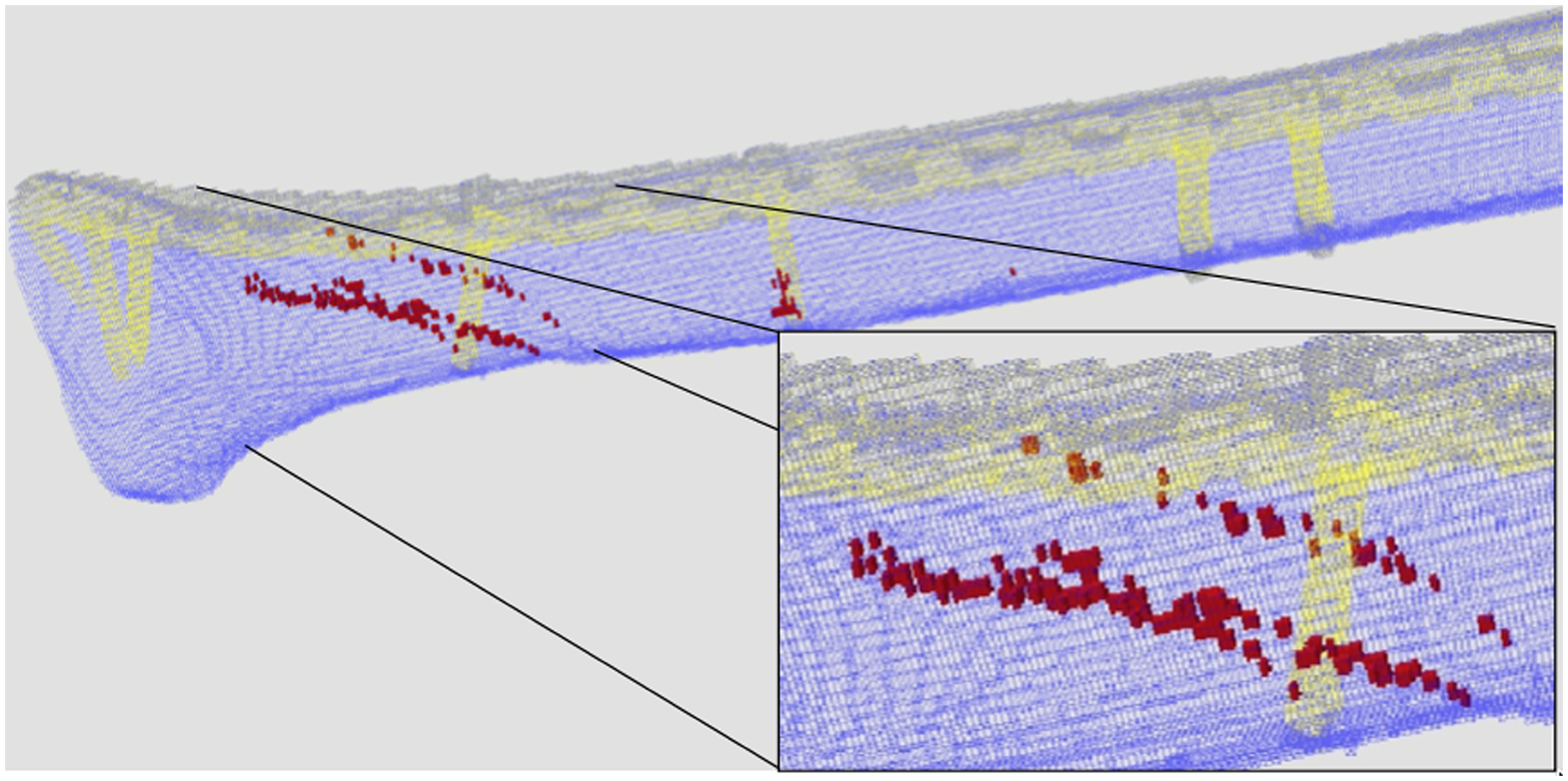

Supplement: Supplementary file 6 — Authors’ original file for figure 6 [file 12891_2014_2362_MOESM6_ESM.tif]
